# An analysis of impact load and fragmentation dimension to explore energy dissipation patterns in coal crushing

**DOI:** 10.1038/s41598-023-45422-7

**Published:** 2023-10-25

**Authors:** Xiao-He Wang, Wu Jing, Wen-Bo Zhang, Jiang-Hao Wang, Qing-Long Yun, Yi-Qing Wang, Sui Yi

**Affiliations:** 1 School of Energy and Mining Engineering, China University of Mining and Technology (Beijing), Beijing, 100083 China; 2 Transportation College, Jilin University, Jilin, 130000 China

**Keywords:** Mineralogy, Petrology

## Abstract

This research delineates the energy dissipation characteristics in coal crushing under impact loads, leveraging the capabilities of Separated Hopkinson Pressure Bar experimental system. A meticulous examination of both burst-prone and non-burst-prone coal samples during destruction processes was undertaken to decipher the dynamic compression mechanical attributes from perspectives of energy and fragmentatio‘s fractal dimensions. Burst-prone coal showcases a more pronounced escalation in fragmentation work in comparison to non-burst-prone samples, thereby illustrating a perceptible strain-rate dependent effect correlating with enhanced strain rates. Additionally, it was observed that incident, reflected, and transmitted energy trajectories for both sample categories follow an approximately linear ascendancy, albeit exhibiting diverse magnitudes. Burst-prone coal manifests a more rapid and focused energy growth compared to its non-burst-prone counterpart. When subjected to impact loads, a notable trend was discerned where the fragmentation’s fractional dimension escalated persistently with both the incident energy and the crushing work, portraying a prominent growth effect. The insights garnered from this study pave the way for distinguishing between impacted and unimpacted coal samples using energy perspectives and fragmentation's fractal dimensions.

## Introduction

As coal mining ventures into greater depths and intensifies, mines characterized by complex geological conditions are witnessing heightened energy accumulation in forefront coal bodies. Mining disturbances can precipitate substantial shock loads, instigated by the abrupt escalation of loads, consequently releasing significant amounts of energy stored over extended periods. This phenomenon stands as a precursor to severe mine catastrophes including, but not limited to, coal and gas outbursts and rock bursts, thereby facilitating considerable economic setbacks and loss of life^1–3^.

Current scholarly endeavors have elucidated disparate mechanical properties and damage modalities in coal rocks under static and dynamic load conditions. The research highlights a pronounced regularity in the damage patterns witnessed under static load conditions^4,5^. Moreover, the fundamental mechanical parameters of dynamic mechanics in coal rocks exhibit a noteworthy strain rate effect under dynamic loading conditions^6^. An extensive body of research underscores the pivotal role of strain rate in altering the properties of coal rocks, thereby affirming its position as a key determinant influencing the shifts in coal rock properties^7,8^. Under the auspices of deep mining conditions, high strain rates potentially instigate augmented impact ground pressure and other profound mine power hazards^9^, thereby posing substantial risks to the safety paradigms in mine operations.

To unravel the mechanical properties of coal rocks under impact loading from a pragmatic standpoint, numerous studies have been conducted utilizing various experimental setups and approaches to dissect the dynamic stress–strain relationships, energy dissipation patterns, and fracture mechanisms involved in coal and rock-like materials. Initiating with the seminal works of Hopkinson^10^ and subsequent enhancements by Kolsky^11^, the experimental apparatus has undergone a series of modifications aimed at controlling air pressure to augment bullet impact velocity, thus facilitating detailed analysis of pulse signals and stress–strain relationships in rock-like materials. Under biaxial static and dynamic coupling conditions, extensive research has been undertaken to understand the coal-breaking process. Noteworthy contributions include investigations by Li^12^ leveraging a triaxial Hopkinson bar to study different impact velocities on coal specimens. Employing Separated Hopkinson Pressure Bar (SHPB) for analysis, Chen^13^ illustrated that increasing strain rates correspond to escalated energy absorption and fragmentation in brittle materials. Pioneers in microscopic analysis, Liu^14^ and Zhan^15^ have detailed the fracturing laws and strain rate's influence on fracture distribution, while Li^16^ examined the evolution of coal rock's pores and fractures under varying impact loads. Further expanding on the subject, an array of scholars has emphasized the positive correlation between strain rate and fragmentation degree in rocks of diverse lithologies^17–20^. Contributing to the discourse, Xie^21,22^ underscored the significant interrelation between rock dissipation energy and porosity, particularly at high strain rates. Building on this, Li^23,24^ inferred a relation between energy dissipation during sandstone damage and different loading approaches, highlighting distinct energy dissipation laws under dynamic and static impacts. Similarly, Jin^25^ explored the influence of temperature on fragmentation, establishing a connection between increasing temperature and escalating energy demands for marble impact damage. Ai^26^ simulated crack propagation and dynamic mechanical properties of coal during SHPB tests, unraveling details of the displacement–strain-stress field. Zhu^27^ predicted rock strength increase factors under various dynamic and static loads through numerical simulations, shedding light on mechanisms governing dynamic rock strength augmentation under combined loads. Wang^28^ embarked on the exploration of mechanical attributes and fracture energy of heterogeneous geomaterials using machine learning techniques for crack classification under dynamic loadings. Further advancements in the field witnessed Ai^29^ proposing an automatic crack detection algorithm to meticulously identify and compute rock surface cracks post SHPB impact loading, while Yang^30^ delved into analyzing the dynamic compression and tensile properties of mortar under impact loading. Zhou^31^ investigated persistent fractured granites, scrutinizing the loading rate effects on dynamic damage and energy evolution during the process. Wu^32^ utilized SHPB to discern the dynamic tensile strength variations of pre-tensioned rocks, with Zhao^33^focusing on the fractal traits of coal-bearing seam crack extensions under similar conditions. Li^34^ entailed introducing radial restraints in SHPB specimens to enhance the compressive strength of concrete-like samples, and Wang^35^ explored the mechanical properties and progressive fracturing of concrete materials under biaxial confinement and repetitive dynamic loads. Xie^36^ ventured into examining the effects of disparate strain rates on the dynamic compression characteristics, energy dissipation, and fragmentation morphology of Basalt Fiber Reinforced Concrete (BFRC). In a foundational study grounded on Separate Hopkins Compression Rod Experiment, Wang^37,38^ embarked on a meticulous exploration to delineate the critical aspects of energy dynamics involved in coal sample impacts, focusing on both burst-prone and non-burst-prone specimens. The comprehensive analysis encompassed the calculations of various energy parameters including incident, reflected, and transmitted energies, in addition to the scrutinizing of crushing work exerted during the impact load. By converging the facets of energy dissipation and strain rate, a pivotal correlation was forged, unveiling a significant link between energy dissipation dynamics and the varying degrees of strain rates. Moreover, leveraging the principles of fractal dimension of fragmentation theory furnished a deeper understanding of the relationship between strain rate and the size of the fragments resulting from the crushing process. A concerted effort to analyze the interrelations among strain rate, crushed particle size, and energy dissipation culminated in the extraction of seminal findings. These encompassed the laws governing energy dissipation and strain rate, alongside insights into the fractal dimensions of fragmentation pertinent to both categories of coal samples. The ramifications of these findings are profound, offering a vital framework for discerning the impactivity of coal samples.

## Methods

### Theoretical study

#### Propagation of stress waves in elastic rods

In scenarios where a previously equilibrated solid medium experiences external disturbances, a breakdown of its original mechanical equilibrium state occurs, thereby triggering substantial alterations in its stress and strain profiles. To thoroughly investigate this phenomenon within the context of an elastic round rod, one can conceptualize the rod as a semi-infinite entity composed of homogenous materials, therein concentrating solely on the axial advancement of the stress wave while dismissing the influences of reflected stress waves for simplicity. In a bid to streamline the analytical process, a key assumption is adopted whereby the cross-section of the deforming elastic rod retains a planar configuration. This approach essentially reduces the issue to a one-dimensional problem space, where the primary focus hinges on analyzing the function delineating the interrelationship between axial stress *x* and time *t*, a function presumed to exhibit uniform distribution along the axial direction.

**Figure 1:**
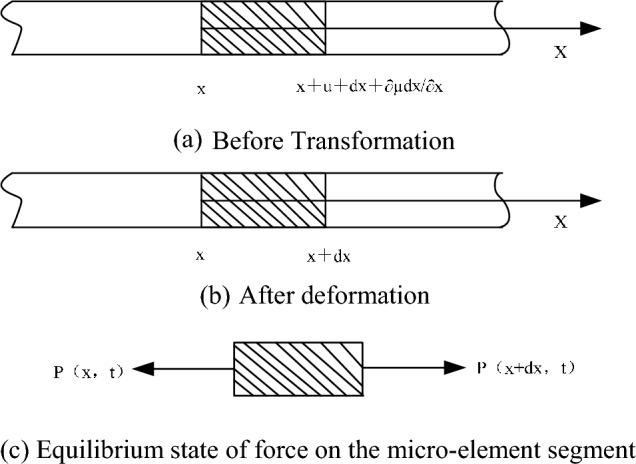
Deformation of microelement in rod.

When an at-rest elastic rod undergoes an applied load *P*, it is essential to dissect the situation by focusing on a diminutive micro-element segment for a detailed force analysis. In this scenario, the x-axis is defined to be aligned with the length of the elastic rod, facilitating a systematic analysis as illustrated in Fig. 1. Here, the initial cross-sectional area and the density of the elastic rod is *A*_0_ and the density is *ρ*_0_. In this scenario, it is pivotal to adhere to Hooke's law which governs the behavior of the small segment under consideration, ensuring an established relationship between the stress and strain induced in the material as follows:

1 $$ \sigma = \frac{P}{{A_{0} }} $$

2 $$ \sigma = E\varepsilon $$

where *σ* and *ε* are the axial stress and strain of the micro-element segment of the elastic rod, respectively; and *E* is the modulus of elasticity of the elastic rod.

In a one-dimensional setting, the displacement *u*, reliant on the spatial position *x* and time *t*, adheres to the continuity conditions. These conditions can be precisely articulated through the continuity equation delineated in Eq. (3):

3 $$ \frac{\partial v}{{\partial x}} = \frac{\partial \varepsilon }{{\partial t}} $$

When the left extremity of the elastic rod is struck suddenly by an applied pressure, it experiences an initial compression concentrated within a narrow region, effectively creating a compressed thin layer. This compressive stress wave subsequently propagates along the rod, transferring force to adjacent thin layers through the rod which serves as the medium for force transmission, a process visually represented in Fig. 1c. Assuming the force exerted on the left cross-section to be *P*(*x, t*), the corresponding force impacting the right cross-section can be formally expressed as follows:

4 $$ P\left( {x + dx,t} \right) = P\left( {x,t} \right) + \frac{{\partial P\left( {x,t} \right)}}{\partial x}dx $$

By invoking Newton’s second law of motion $F = ma$, the stress wave equation of motion is obtained as follows:

5 $$ \rho_{0} \frac{\partial v}{{\partial t}} = \frac{\partial P}{{\partial x}} $$

The wave velocity in the one-dimensional state can be defined as follows:

6 $$ C_{0} = \sqrt {\frac{E}{{\rho_{0} }}} $$

The linear fluctuation equation is obtained by substituting Eq. (6) into Eq. (5):

7 $$ \frac{{\partial^{2} u}}{{\partial x^{2} }} = C_{0}^{2} \frac{{\partial^{2} u}}{{\partial x^{2} }} $$

In reality, this formulation operates under a one-dimensional assumption, sidelining the potential transverse movements occurring during the impact on rod masses. This approximation holds provided the stress wave's wavelength within the rod markedly exceeds the rod's transverse dimension. Given a consistent loading process and a uniform wave speed, it can be ensured that the waveform retains its stability as it traverses through the elastic rod; this implies that under one-dimensional conditions, the stress wave does not undergo dispersion throughout its propagation in the elastic rod.

#### One-dimensional collision of two elastic rods

**Figure 2:**
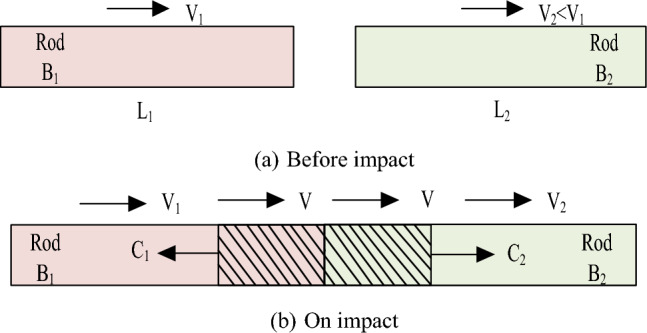
Impact of two elastic rods.

The foundational representation of the separated compression bar experimental apparatus involves the interaction between two elastic rods during an impact event. Assign labels B_1_ and B_2_ to these rods, which are characterized by identical cross-sectional dimensions. Let the wave impedances associated with the stress compression waves in each rod be $\rho_{1} C_{1}$ and $\rho_{2} C_{2}$, and their initial velocities of mass movement be $v_{1}$ and $v_{2}$, with the condition $v_{1} > v_{2}$. t the outset, both rods are devoid of any stress, a state depicted in Fig. 2a. Upon collision, a compression wave of stress is initiated at their contact interface. This wave exhibits a unidirectional propagation from the left to the right, maintaining a consistent speed, a phenomenon illustrated in Fig. 2b.

Under the presumption that the two elastic rods attain a common wave velocity *v* post-impact, one can invoke the principles of continuity and Newton's third law. These principles dictate that, during the impact phase, the masses situated at the respective cross-sections of the rods achieve a uniform velocity. Simultaneously, the stresses associated with the propagating compressional waves equilibrate. This leads to formulate the following relationships:

8 $$ \sigma = \rho_{1} C_{1} \left( {v - v_{1} } \right) = \rho_{2} C_{2} \left( {v - v_{2} } \right) $$

Given a scenario where the wave impedances of the two elastic rods are equal, prepresented as $\rho_{1} C_{1} = \rho_{2} C_{2} = \rho_{3} C_{0}$, this equality allows for substantial simplification in the equations as follows:

9 $$ v = \frac{1}{2}\left( {v_{1} + v_{2} } \right) $$

10 $$ \sigma = - \frac{1}{2}\rho_{0} C_{0} \left( {v_{1} - v_{2} } \right) $$

In the foregoing analysis, the focus has been solely on the shock compression wave generated by a single impact, while disregarding lateral inertial forces and potential reflections occurring at the rod ends^39^.

Leveraging the principles of continuity and Newton's third law facilitates the understanding as follows:

11 $$ \sigma_{3} = - \rho_{0} C_{0} v_{1} + \rho_{0} C_{0} \left( {v_{3} - v_{1} } \right) = - \rho_{0} C_{0} \left( {v_{3} - v_{2} } \right) $$

From the derived equation above, the stress and velocity vectors of the two elastic rods can undergo superposition in the post-impact stress wave scenario.

12 $$ v_{3} = v_{1} + v_{2} $$

13 $$ \sigma_{3} = \sigma_{1} + \sigma_{2} $$

From the derived equation above, the stress and velocity vectors of the two elastic rods can undergo superposition in the post-impact stress wave scenario.

Figure 2 designates the length of the shorter rod B_1_ as L_1_ and that of the longer rod B_2_ as L_2_. In the event where the shorter rod B_1_ impacts the longer rod B_2_ due to an applied force, a primary elastic stress wave is initiated, traversing from B_1_ towards B_2_. This transmission persists until time *t* satisfies the condition $t = L_{1} {/}C_{1}$, at which point the wave encounters its first reflection at the left extremity of rod B_2_, engendering a secondary elastic wave that continues to propagate along the rod's longitudinal axis. As time progresses to $t = L_{1} {/}C_{1}$, a phenomenon occurs where the wave undergoes a reflection at the right interface of rod B_2_, transforming into a tensile stress wave that operates to negate the influence of the preceding incident stress wave. A detailed exploration concerning the varying wave impedances of the elastic rods follows in the subsequent sections.

1. When $\rho_{1} C_{1}$ = $\rho_{2} C_{2}$, the wave impedances of the short rod B_1_ and the long rod B_2_ are equal. Under these conditions, the two elastic rods remain in a state of compression during the impact. The stress fluctuations that arise in this situation have the potential to be transformed into strain energy. Furthermore, the reflected stress waves, which originate from the contact surface where B_1_ and B_2_ meet, transmit seamlessly along the trajectory of the long rod, thus avoiding unnecessary reflections.
2. When $\rho_{1} C_{1}$ > $\rho_{2} C_{2}$, the wave impedance of the short rod B_1_ exceeds that of the long rod B_2_, a particular dynamic unfolds during the collision process. As B_1_ engages in an impact with B_2_, a consequent reflection occurs at the right free end of B_1_, leading to a progressive diminution in both the velocity and stress of the stress wave. Moreover, when the secondary elastic stress wave—originating from the left free end of B_2_—is reflected back, there is a gradual mutual annihilation of the stress waves. This interaction navigates towards a state where the stress decrement approximates zero, thus delineating a pathway to a cessation in the stress activity.
3. When $\rho_{1} C_{1}$ < $\rho_{2} C_{2}$, a dynamic characterized by the lesser wave impedance of short rod B1 relative to that of long rod B_2_ unfolds. Following the initiation of the elastic stress wave at the right free extremity of B1, there is a propagation along the axis of B_2_, a trajectory punctuated by several reflections that culminate in a cessation of stress as it attenuates to zero. During this phase, when $t = 2L_{1} {/}C_{1}$, a phenomenon occurs: a reversal in mass velocity to a negative value is observed. This sets the stage for a subsequent event where B_2_, now with a velocity 2v_3_, impinges upon B_1_ post-displacement. This engagement results in only a fraction of the stress oscillations in B_1_ being relayed to B_2_. The majority of the momentum, rather than being transferred, experiences a reflection at B_2_'s free extremity, illustrating a significant retention of kinetic energy within the system.

#### Reflected and transmitted stress waves in elastic rods

Upon the collision of two elastic rods, an emergent elastic stress wave navigates through the distinct mediums of each rod during its propagation journey. This complex process involves entering from one medium to another; a transition accompanied by notable phenomena when the wave impedances of these mediums differ. Under such circumstances, the elastic stress wave experiences simultaneous reflection and transmission at the interface of the two disparate media surfaces.

**Figure 3:**
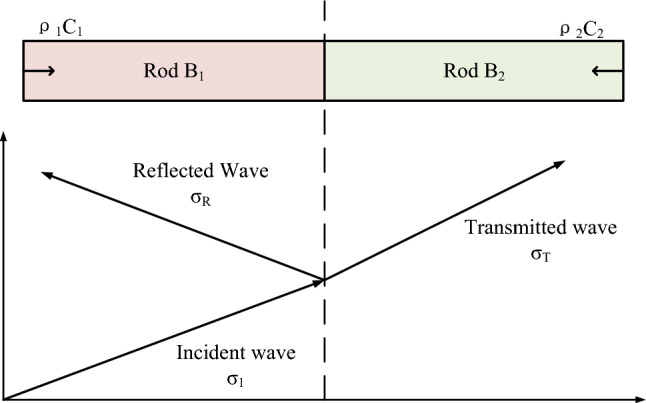
Reflection and transmission of elastic waves at different media interfaces.

As illustrated in Fig. 3, two homogeneous elastic rods with identical cross-sectional diameters are under consideration. The short rod B_1_ has a wave impedance denoted as $\rho_{1} C_{1}$, and the long rod B_2_ has a wave impedance characterized by $\rho_{2} C_{2}$. Upon impact, an elastic stress incident wave $\sigma_{1}$, propagates from the short rod B_1_ towards the long rod B_2_. This propagation reaches a critical point at the contact section between the two rods at $\rho_{1} C_{1}$. At this junction, the unbounded end of the long rod becomes a source point for two simultaneous phenomena: the emergence of a reflected stress wave $\sigma_{R}$ and a transmitted stress wave $\sigma_{T}$.

As the incident wave reaches the juncture where the two different media characterized by $\rho_{2} C_{2}$ intersect, a pivotal transformation occurs. Here, the incident wave undergoes both reflection and transmission, giving rise to a complex interplay of waves at the intersection. Leveraging Newton's third law alongside the principle of mass continuity facilitates deriving critical relationships regarding this dynamic system. A foundational premise emerges—post reflection and transmission, the velocity and stress governing the propagating masses should align, maintaining a state of equilibrium, articulated as follows:

14 $$ v_{1} + v_{R} = v_{T} $$

15 $$ \sigma_{1} + \sigma_{R} = \sigma_{T} $$

where $v_{1}$, $v_{R}$, and $v_{T}$ are the mass velocities of the incident, reflected, and transmitted waves, respectively; $\sigma_{1}$, $\sigma_{R}$, and $\sigma_{T}$, the stresses of the incident, reflected, and transmitted waves, respectively.

The velocity increments of the incident, reflected, and transmitted waves can also be expressed as follows:

16 $$ v_{1} = \frac{{\sigma_{1} }}{{\rho_{1} C_{1} }},v_{R} = \frac{{\sigma_{R} }}{{\rho_{1} C_{1} }},v_{T} = \frac{{\sigma_{T} }}{{\rho_{2} C_{2} }} $$

$$ \sigma_{R} = \frac{{\rho_{1} C_{1} - \rho_{2} C_{2} }}{{\rho_{1} C_{1} + \rho_{2} C_{2} }},\,\,\,v_{R} = \frac{{\rho_{1} C_{1} - \rho_{2} C_{2} }}{{\rho_{1} C_{1} + \rho_{2} C_{2} }}v_{1} $$

17 $$ \sigma_{T} = \frac{{2\rho_{1} C_{1} }}{{\rho_{1} C_{1} + \rho_{2} C_{2} }}\sigma_{1} ,\,\,\,\,v_{T} = \frac{{2\rho_{1} C_{1} }}{{\rho_{1} C_{1} + \rho_{2} C_{2} }}v_{1} $$

Assuming $\beta = \rho_{1} C_{1} /\rho_{2} C_{2}$ be the ratio of wave impedance of two dielectric materials, it can be defined as follows:

18 $$ F = \frac{{\rho_{1} C_{1} - \rho_{2} C_{2} }}{{\rho_{1} C_{1} + \rho_{2} C_{2} }} = \frac{1 - \beta }{{1 + \beta }} $$

19 $$ T = \frac{{2\rho_{1} C_{1} }}{{\rho_{1} C_{1} + \rho_{2} C_{2} }} = \frac{2}{1 + \beta } $$

where $F$ and $T$ are the reflection system and transmission coefficient, respectively. The relationship can be expressed as follows:

20 $$ 1 + F = T $$

Substituting Eqs. (18) and (19) into Eqs. (16) and (17), these parameters can be determined as follows:

21 $$ \sigma_{R} = F\sigma_{1} ,\,\,\,v_{R} = - Fv_{1} $$

22 $$ \sigma_{T} = T\sigma_{1} ,\,\,\,\sigma_{R} = - \beta Tv_{1} $$

Through a meticulous analysis of the wave impedance characteristics inherent to distinct media materials involved in the interaction, it becomes feasible to delineate the transmission coefficient $T$. This coefficient can be mathematically established as maintaining a constant positive value. Conversely, the reflection coefficient $F$, exhibits a range of values, encapsulating both positive and negative magnitudes. This dichotomy necessitates a thorough exploration of the diverse scenarios that may transpire, leading to classify the potential occurrences into three distinct categories for systematic discussion and analysis:

1. When $\beta < 1$ and $\rho_{1} C_{1} < \rho_{2} C_{2}$, the wave impedance of medium 1 is smaller than the wave impedance of medium 2, indicating that the reflected wave and stress wave exhibits same sign; when $T > 1$, the stress amplitude of the incident wave is smaller than the transmitted wave; when $\rho_{2} C_{2} \to \infty \left( {\beta \to 0} \right)$, $F = 1$ and $T = 2$.
2. When $\beta > 1$ and $\rho_{1} C_{1} > \rho_{2} C_{2}$, the wave impedance of medium 1 surpasses that of medium 2, a discernible disparity arises in the magnitudes of the reflected wave and the incident stress wave. This phenomenon culminates in a scenario wherein the incident and reflected waves engage in a mutual negation, canceling each other out; when $T < 1$, the stress amplitude of the incident wave surpasses that of the transmitted wave; when $\rho_{2} C_{2} \to 0\left( {\beta \to \infty } \right)$, at this time $F = - 1$ and $T = 0$.
3. When $\beta = 1$ and $\rho_{1} C_{1} = \rho_{2} C_{2} = \rho_{0} C_{0}$, the wave impedance of medium 1 precisely aligns with that of medium 2, an optimal condition is realized whereby the stress wave seamlessly transmits across the boundaries of the two mediums without any reflection occurring. This situation, which can be considered ideal, is actively sought in SHPB experiments. Ensuring identical wave impedance across differing media surfaces eradicates any potential reflection at the juncture of the two interfaces, thereby facilitating unimpeded transmission through the intersection. This not only obviates dispersion phenomena that can occur during the propagation of the elastic stress wave but also significantly mitigates the margin of experimental error, promising more accurate, reliable results.

### Configuration and fundamental operations of SHPB experimental device system

**Figure 4:**
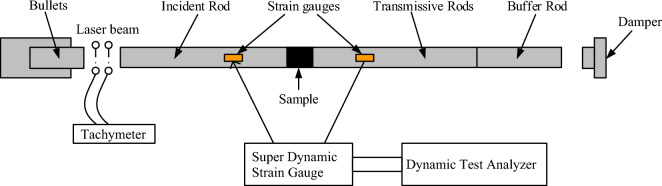
SHPB experimental system.

**Figure 5:**
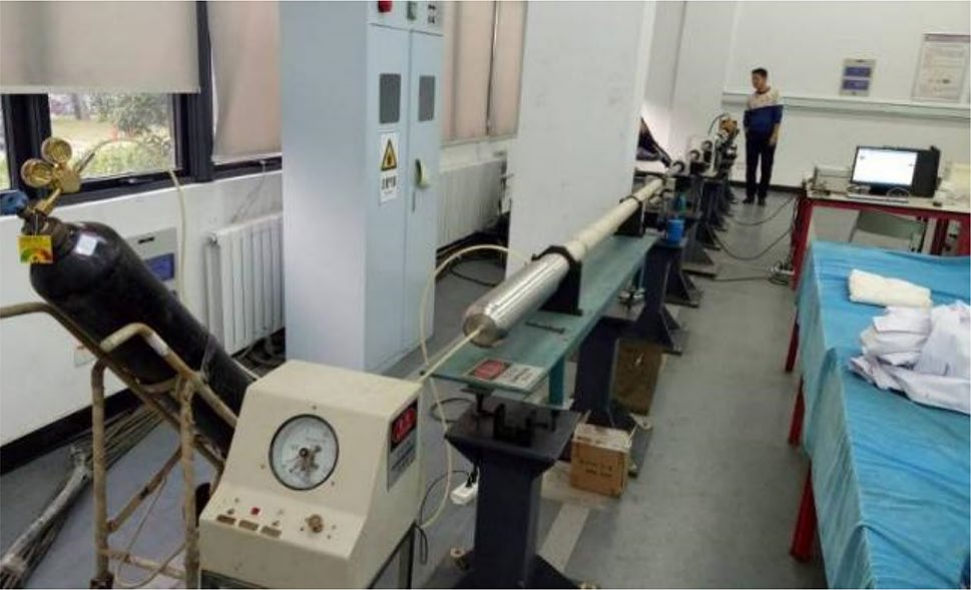
Testing workflow overview.

SHPB experimental system is composed of several integral components which include a power loading driver, leveraged through a high-pressure N cylinder, a firing chamber, and a short impact bar—fabricated from Cr alloy to maintain rigidity. This latter component functions as a bullet device within the setup. Essential measurement tools integrated into the system comprise a strain gauge, a velocimeter, and a dynamic test analyzer, facilitating precise data acquisition during experiments. A detailed breakdown of the system reveals an incidence bar (also referred to as the input bar) and a transmission bar (or output bar), both crafted from the same material and possessing identical dimensions to ensure uniformity and reliability in performance. The apparatus is designed to maintain a harmonious operational flow, effectively illustrating the dynamics of material behavior under pressure. To provide visual aids for better comprehension, schematic representations of the experimental system alongside real-time field test diagrams are depicted in Figs. 4 and 5.

In pursuit of accurately delineating the inherent mechanical attributes of coal rocks in dynamic settings, SHPB experimental testing technique is grounded on two assumptions:

1. The strategy essentially overlooks the influences wielded by the strain rates of the materials constituting the elastic and reflective rods during the experiment. It envisions the compressive stress wave embarking on a one-dimensional trajectory in both rods, immune to dispersion effects. Furthermore, it stipulates that the strains enacted in every direction of the compressive stress waves within this one-dimensional stress realm resonate uniformly with the end facets of the engaged specimens. This facilitates a straightforward extrapolation of the coal specimens' stress strain through the application of rudimentary one-dimensional theory.
2. It presumes a uniform variation in the stress strain along the specimen’s longitudinal axis over truncated time intervals, effectively sidelining the repercussions of the specimen's compressive stress wave dynamics.

For hypothesis (1), the objective is to facilitate the one-dimensional propagation of the elastic stress wave through the rod. This necessitates employing a compression bar of reduced diameter, tasked with generating an incident elastic stress wave characterized by a sine wave profile. This configuration is within the realm of feasibility given the controlled experimental settings.

For hypothesis (2), as the elastic stress wave navigates towards the specimen, a crucial determinant in this stage is the acoustic impedance, which can be defined as follows:

23 $$ \left\{ {\begin{array}{*{20}c} {\beta_{0} = \rho_{0} C_{0} A_{0} } \\ {\beta_{s} = \rho_{s} C_{s} A_{s} } \\ \end{array} } \right. $$

where $\beta_{s}$, $\rho_{s}$, $C_{s}$, and $A_{s}$ denote the acoustic impedance, density, stress wave velocity, and cross-sectional area of the compressional rod material, respectively.

When the elastic stress wave reaches the juncture where the compression bar and the specimen intersect, the coefficients corresponding to reflection and transmission can be defined as follows:

24 $$ \left\{ {\begin{array}{*{20}l} {\alpha_{1} = \frac{{\beta_{s} - \beta_{0} }}{{\beta_{s} + \beta_{0} }},\gamma_{1} = \frac{{A_{s} }}{{A_{0} }}\frac{{2\beta_{s} }}{{\beta_{s} + \beta_{0} }}} \hfill \\ {\alpha_{2} = \frac{{\beta_{0} - \beta_{s} }}{{\beta_{s} + \beta_{0} }},\gamma_{1} = \frac{{A_{0} }}{{A_{s} }}\frac{{2\beta_{0} }}{{\beta_{s} + \beta_{0} }}} \hfill \\ \end{array} } \right. $$

where $\alpha_{1}$ and $\alpha_{2}$ are reflection coefficients; and $\gamma_{1}$ and $\gamma_{2}$ are pair transmission coefficients.

As the elastic stress wave transitions from the specimen to the transmission rod, the stress manifests as follows:

25 $$ \sigma_{3} = \sigma_{1} \gamma_{1} \gamma_{2} = \sigma_{1} \left[ {1 - \left( {\frac{{\beta_{0} - \beta_{s} }}{{\beta_{0} + \beta_{s} }}} \right)^{2} } \right] $$

By utilizing analogous reasoning, the following is derived:

26 $$ \left\{ {\begin{array}{*{20}l} {\sigma_{5} = \sigma_{1} \left[ {1 - \left( {\frac{{\beta_{0} - \beta_{s} }}{{\beta_{0} + \beta_{s} }}} \right)^{4} } \right]} \hfill \\ {\sigma_{7} = \sigma_{1} \left[ {1 - \left( {\frac{{\beta_{0} - \beta_{s} }}{{\beta_{0} + \beta_{s} }}} \right)^{6} } \right]} \hfill \\ \ldots \hfill \\ {\sigma_{2n + 1} = \sigma_{1} \left[ {1 - \left( {\frac{{\beta_{0} - \beta_{s} }}{{\beta_{0} + \beta_{s} }}} \right)^{2n} } \right]} \hfill \\ \end{array} } \right. $$

When $\beta_{0} < \beta$ and $n \to \infty$, it can be determined as follows:

27 $$ \mathop {lim}\limits_{n \to \infty } \sigma_{2n + 1} = \sigma_{1} $$

An analysis of Eqs. (26) and (27) reveals that, following several reflections of the elastic stress wave at the interface between the specimen and the two elastic rods, there occurs a redistribution of stresses within the specimen, ultimately stabilizing into a condition of stress equilibrium.

### Test principle of SHPB experimental device system

**Figure 6:**
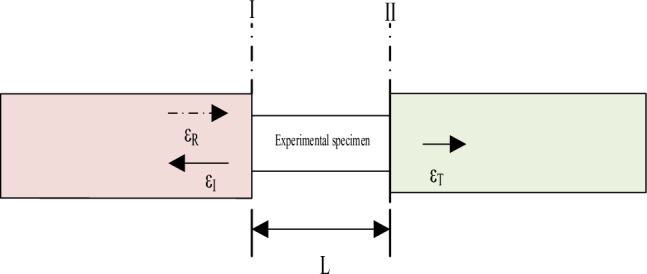
SHPB system schematic.

In Fig. 6, $\varepsilon_{I}$ is the incident signal; $\varepsilon_{R}$ is the reflected signal; $\varepsilon_{T}$ is the transmitted signal; $A_{s}$ is the cross-sectional area of the specimen; I and II represent the two end surfaces of the specimen; and L is the length of the specimen. The total displacement experienced by the elastic rod can be delineated using the principles outlined in the one-dimensional stress wave propagation theory, wherein the specifics are detailed below:

28 $$ u = C_{s} \mathop \smallint \limits_{0}^{t} \varepsilon dt $$

In Eq. (1), the displacements of the two end faces of the specimen can be determined as follows:

29 $$ u_{1} = \mathop \smallint \limits_{0}^{t} C_{0} \varepsilon_{1} dt $$

30 $$ u_{2} = \mathop \smallint \limits_{0}^{t} C_{0} \varepsilon_{2} dt $$

Therefore, the displacement $u_{1}$ on interface 1 can be expressed as follows:

31 $$ u_{1} = C_{0} \mathop \smallint \limits_{0}^{t} \left( {\varepsilon_{I} - \varepsilon_{R} } \right)dt $$

The displacement $u_{2}$ at interface 2 is only related to the transmission pulse $\varepsilon_{T}$, which can be calculated as follows:

32 $$ u_{2} = C_{0} \mathop \smallint \limits_{0}^{t} \varepsilon_{T} dt $$

The average strain of the incident, reflected, and transmitted strain with signals of the specimen can be expressed as follows:

33 $$ \varepsilon_{s} = \frac{{C_{0} }}{L}\mathop \smallint \limits_{0}^{t} (\varepsilon_{I} - \varepsilon_{R} - \varepsilon_{T} )dt $$

The applied forces on the terminating faces of specimens 1 and 2 can be discerned through the application of one-dimensional elastic theory, as delineated below:

34 $$ P_{1} \left( t \right) = EA\left[ {\varepsilon_{I} \left( t \right) + \varepsilon_{R} \left( t \right)} \right] $$

35 $$ P_{2} \left( t \right) = EA\varepsilon_{T} \left( t \right) $$

In summary, the equations pertinent to the three-wave methodology in SHPB experimental measurements can be delineated as follows:

36 $$ \left\{ {\begin{array}{*{20}l} {\sigma_{s} \left( t \right) = \frac{{P_{1} \left( t \right) + P_{2} \left( t \right)}}{{2A_{s} }} = \frac{EA}{{2A_{s} }}\left[ {\varepsilon_{I} \left( t \right) + \varepsilon_{R} \left( t \right) + \varepsilon_{T} \left( t \right)} \right]} \hfill \\ {\varepsilon_{s} \left( t \right) = \frac{{C_{0} }}{{I_{s} }}\mathop \smallint \limits_{0}^{t} \left[ {\varepsilon_{I} \left( t \right) - \varepsilon_{R} \left( t \right) - \varepsilon_{T} \left( t \right)} \right]} \hfill \\ {\dot{\varepsilon }_{s} \left( t \right) = \frac{{C_{0} }}{{I_{s} }}\left[ {\varepsilon_{I} \left( t \right) - \varepsilon_{R} \left( t \right) - \varepsilon_{T} \left( t \right)} \right]} \hfill \\ \end{array} } \right. $$

Assuming uniformity, wherein the cross-sectional areas of the two rods are equivalent, it follows from the principle of homogeneity that $F_{1} = F_{2}$, denoted as follows:

37 $$ \varepsilon_{I} + \varepsilon_{R} = \varepsilon_{T} $$

By incorporating Eq. (37) into Eq. (36), the dual-wave formulation pertinent to SHPB experimental assessments can be derived as follows:

38 $$ \left\{ {\begin{array}{*{20}l} {\sigma_{s} \left( t \right) = \frac{EA}{{A_{s} }}\varepsilon_{T} \left( t \right)} \hfill \\ {\varepsilon_{s} \left( t \right) = - 2\frac{{C_{0} }}{L}\mathop \smallint \limits_{0}^{t} \varepsilon_{R} \left( t \right)dt} \hfill \\ {\dot{\varepsilon }_{s} \left( t \right) = - 2\frac{{C_{0} }}{L}\varepsilon_{R} \left( t \right)} \hfill \\ \end{array} } \right. $$

### Specimen preparation and testing procedure

In this experiment, a comprehensive selection of 36 coal specimens was undertaken, derived from Xin Zhou Yao mine (without bursts) and Ma Chang mine (with bursts). These specimens were designated to explore the dissipation dynamics of coal rocks under conditions of impact loading. The selection encompassed six distinct groups, evenly bifurcated between burst-prone and non-burst-prone coal samples, each containing three specimens, maintaining dimensions of φ 50 × 50 mm. The meticulous preparation procedure began with the extraction of uniform diameter coal samples utilizing a coring machine. Following this, both the extremities were precisely sheared and polished employing a cutting and grinding apparatus, respectively. This ensured the attainment of stringent tolerance levels in flatness (< 0.5 mm) and parallelism (< 0.02 mm) as necessitated by the experimental parameters, thereby validating the readiness of the specimens for the ensuing assessments.

The examination of dynamic mechanical attributes of coal rocks unfolds primarily through the successive steps delineated below: Initially, coal specimens are meticulously positioned between the incident (input) and transmission (output) rods. To mitigate the potential interference of transverse strain induced by the stress wave during the impact, a coating of petroleum jelly reagent is applied to foster optimal coupling between the specimen and the dual elastic rods. Subsequently, liquid N serves the pivotal role of delivering the requisite load at the impact terminal, with the magnitude of the air pressure meticulously regulated to govern the incident stress wave's propagation dynamics at the junction of the specimen and incident rod. In the ensuing phase, this stress wave undergoes reflection and transmission, giving rise to concurrent electrical signals. This necessitates the strategic affixing of strain gauges at both extremities of the incident and transmitted rods, thereby facilitating the comprehensive capture and transmission of all electrical signals to the data acquisition system. Drawing upon the acquired pulse signals of both the incident and reflected waves, alongside the transmitted waves, a deeper analytical endeavor allows for the extraction of the stress–strain curve, effectively charting the dynamic load-induced mechanical properties of the specimen. The aforementioned procedures, when executed with precision, culminate in the derivation of the stress–strain curve, offering a detailed portrayal of the specimen's mechanical responsiveness under dynamic loading conditions.

## Results

### Energy composition in SHPB compression bar experiment

In the context of SHPB compression bar experiment, a comprehensive analysis of energy distribution plays a pivotal role in understanding the dynamics at play. The system chiefly involves five categories of energy: the energy originating from bullet impact, the incident energy *W*_*I*_, the reflected energy *W*_*R*_, the transmitted energy *W*_*T*_, and the dissipated crushing work *W*_*L*_. Each component has a unique role: the bullet impact energy is the initial energy transferred to the incident bar at the point of collision; the incident energy, denoted as *W*_*I*_, is the energy conveyed through the incident stress wave as it propagates through the incident bar; the reflected energy *W*_*R*_ is the part of the incident energy reflected back upon encountering the sample; the transmitted energy *W*_*T*_ denotes the energy fraction that passes through the sample to reach the transmission bar; finally, the dissipated crushing work *W*_*L*_ pertains to the energy absorbed by the sample, which accounts for deformation and fragmentation processes. This energy landscape in SHPB experiment is underpinned by the principles of elastic wave theory, which posits that during the impact damage phase involving the coal sample, the energy introduced by the bullet divides as it meets the sample, with portions being reflected and transmitted through different pathways. This theoretical background allows for the mathematical representation of the energies of the incident, reflected, and transmitted stress waves in the compression bar system, to be illustrated as expressed in Eq. (39):

39 $$ \left\{ {\begin{array}{*{20}l} {W_{I} = \frac{AC}{E}\mathop \smallint \limits_{0}^{t} \sigma_{i}^{2} \left( t \right)dt = ACE\mathop \smallint \limits_{0}^{t} \varepsilon_{i}^{2} \left( t \right)dt} \hfill \\ {W_{R} = \frac{AC}{E}\mathop \smallint \limits_{0}^{t} \sigma_{r}^{2} \left( t \right)dt = ACE\mathop \smallint \limits_{0}^{t} \varepsilon_{r}^{2} \left( t \right)dt} \hfill \\ {W_{T} = \frac{AC}{E}\mathop \smallint \limits_{0}^{t} \sigma_{t}^{2} \left( t \right)dt = ACE\mathop \smallint \limits_{0}^{t} \varepsilon_{t}^{2} \left( t \right)dt} \hfill \\ \end{array} } \right. $$

where $W_{I}$, $W_{R}$, and $W_{T}$ are the incident energy, reflected energy, and transmitted energy, respectively; $\sigma_{i}$, $\sigma_{r}$, and $\sigma_{t}$ are the stresses corresponding to the incident, reflected, and transmitted waves on the compression bar, respectively; $\varepsilon_{i}$, $\varepsilon_{r}$, and $\varepsilon_{t}$ are the strains corresponding to the stresses of each stress wave on the compression bar; *A* is the cross-sectional area of the impact bar and the output bar, (i.e., $A = \pi r^{2}$, where r = 25 mm; *E* is the compression bar E is the elastic modulus of the material, (210 GPa); *C* is the wave velocity of the stress wave in the one-dimensional state, which can be expressed as $C = \sqrt {E/\rho }$, here $\rho$ is the material density of the compression bar and $\rho_{0}$ = $7.8 \times 10^{3}$ kg/m^3^; this SHPB experimental system utilizes *C* value of 5190 m/s.

Throughout the duration of SHPB experiment, a petroleum jelly lubricant was applied to both extremities of the coal sample, facilitating the assumption that any frictional resistance between the compression rod and the coal specimen can be disregarded. This means that no energy will be depleted due to friction between the compression rod and the coal sample. Leveraging the principles encapsulated in the law of conservation of mass, the energy dissipated over the course of the coal sample's degradation—referred to as the crushing work *W*_*L*_—can be articulated as follows:

40 $$ W_{L} = W_{I} - \left( {W_{R} + W_{T} } \right) = ACE\mathop \smallint \limits_{0}^{t} \left\{ {\varepsilon_{i}^{2} \left( t \right) - \left[ {\varepsilon_{r}^{2} \left( t \right) + \varepsilon_{t}^{2} \left( t \right)} \right]} \right\}dt $$

Given the destructive mechanics scrutinized in SHPB experiments with coal samples, when these samples undergo impact tests, the work involved in the fragmentation of the coal encompasses energy dissipation distributed across three distinct categories: (1) Energy expended facilitating the expansion and rupture of intrinsic fractures as well as cultivating new microfractures within the samples; (2) Kinetic energy utilized when the samples fracture, leading to the dispersion of fragmented pieces; and (3) Energy largely designated for alternative modes of heat dissipation including, but not limited to, acoustic energy, thermal energy, and the generation of frictional heat. Insight derived from Hong Liang's exploration^40^ of rock mechanical properties through SHPB experimental framework posits that an overarching majority of the energy, exceeding 95%, primarily serves the extension of existing fractures and the inception of new ones, guiding the specimen to its eventual breaking point, whereas less than 5% of the energy finds its use in other heat dissipation avenues.

The dynamic compression trials facilitated the acquisition of stress–strain curves pertinent to both impacted and non-impacted coal samples. Leveraging Eq. (39), the harvested data from these trials were meticulously integrated to unravel the underlying principles governing the fluctuating tendencies of various energy components—including incident energy *W*_*I*_, reflected energy *W*_*R*_, transmitted energy *W*_*T*_, and the energy associated with crushing work *W*_*L*_—in correlation with the strain rate, a comprehensive detailing of which has been tabled in Table 1.

**Table 1:** Energy variation versus strain rate in coal sample destruction.

Coal sample type	No	Average incident velocity/(m/s)	Strain rate $\dot{\varepsilon }/s^{ - 1}$	Mean value of incident energy/J	Mean value of reflected energy/J	Mean value of transmissive energy/J	Mean value of breaking work/J
Burst-prone coal sample	X-1	6.31	87.76	73.65	62.56	1.26	9.83
	X-2	8.07	94.32	92.45	70.05	1.42	20.68
	X-3	8.71	115.17	111.73	84.21	1.61	25.91
	X-4	9.72	125.26	134.28	90.78	1.85	41.65
	X-5	10.81	146.67	226.47	166.02	1.96	58.49
	X-6	11.64	168.83	269.56	184.97	2.15	82.45
Non-burst-prone coal sample	Y-1	6.39	67.43	41.74	35.44	0.85	5.45
	Y-2	7.97	80.37	63.51	53.12	0.94	9.45
	Y-3	8.77	124.45	176.38	127.32	1.13	47.93
	Y-4	9.75	139.39	241.45	183.10	1.26	57.09
	Y-5	10.87	160.27	284.37	213.98	1.37	69.42
	Y-6	11.7	220.28	471.43	392.05	1.48	77.90

### Effect of impact velocity on stress wave energy

**Figure 7:**
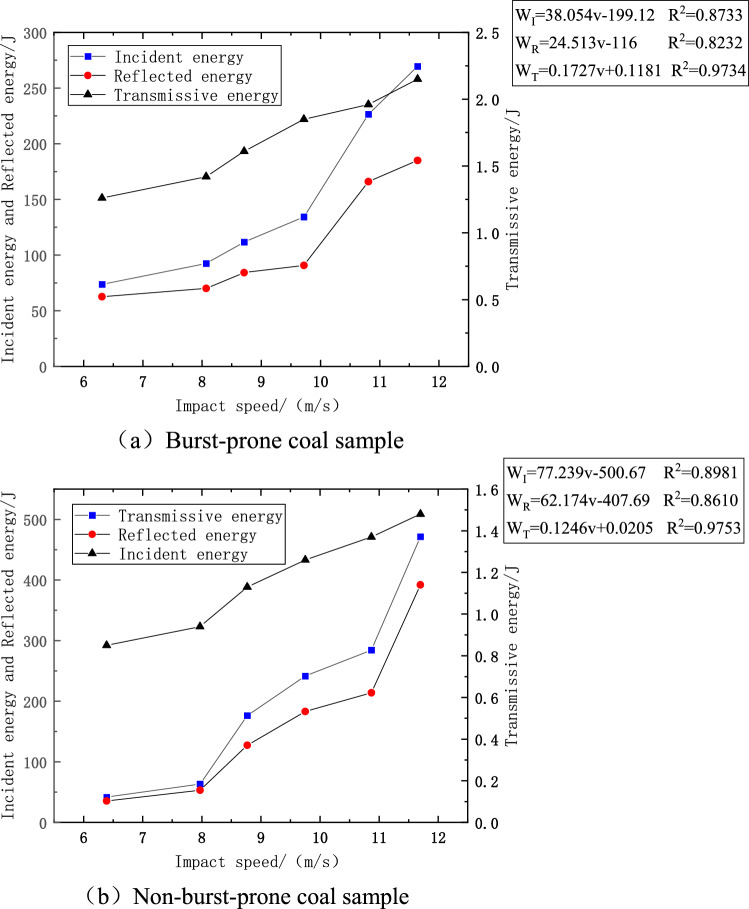
Relationship between energy of stress waves and impact velocity.

Figure 7 delineates the relationship between various stress wave energies and impact velocity, as observed in two distinct sets of specimens. It can be discerned that both the incident and reflected energies exhibit a pronounced increment, commensurate with the rise in impact velocity, a trend that is prevalent in both specimen groups. The modulation of impact pressure between 0.200 and 0.325 MPa, achieved through the calibrated deployment of liquid N, facilitated the control of impact velocities, thereby enabling the application of varied strength loads on the subjects of the experiment. As the impact velocity escalates, a conspicuous trend emerges, revealing a more rapid augmentation in the energy parameters for the impacted specimen during the early stages compared to the non-impacted one. This can potentially be attributed to the predisposition of the initially impacted coal sample to foster energy concentration zones more readily with escalating velocities, thus manifesting larger values of incident and reflected energies initially. Further observation underscores a significant phenomenon occurring at an impact velocity threshold of 8.71 m/s, beyond which both the incident and reflected energies in the non-impacted specimen delineate an exponential surge. This infers the existence of minuscule fractures within the specimen, which necessitate a span for closure under the onslaught of impact pressure. However, an increase in impact velocity diminishes this time frame, inciting a marked rise in the respective energies.

Analysis of the data presented in Fig. 7 indicates a steady state of the curve correlation coefficient R^2^, which has stabilized around the value of 0.9, demonstrating data stability and a high degree of reliability and authenticity. Figure 7a portrays a significant escalation in the different energy forms observed in the impact specimen. A detailed analysis delineates a substantial increase in incident energy from 73.65 to 269.56 J, marking a 366% increment year-on-year. Similarly, the reflected energy observed a growth from 62.56 to 184.97 J, registering a 296% increment year-on-year. Furthermore, the transmittance energy displayed a growth, albeit at a lesser magnitude, increasing from 1.26 to 2.15 J, which translates to a 170% increase year-on-year. On inspecting Fig. 7b, it becomes evident that the no-impact specimen underwent substantial energy alterations, with the incident energy shooting up from 41.74 to a remarkable 471.43 J, an augmentation amounting to 1129% year-on-year. This trend is mirrored in the reflected energy as well, which saw an elevation from 35.44 to 392.05 J, indicating an increment of 1106% year-on-year. Even the transmitted energy observed a surge from 0.85 to 1.48 J, marking a 174% increase year-on-year. This analytical scrutiny unequivocally substantiates a significant amplification in the stress wave energy parameters in concurrence with the escalation in impact velocity.

### Effect of impact velocity on strain rate

**Figure 8:**
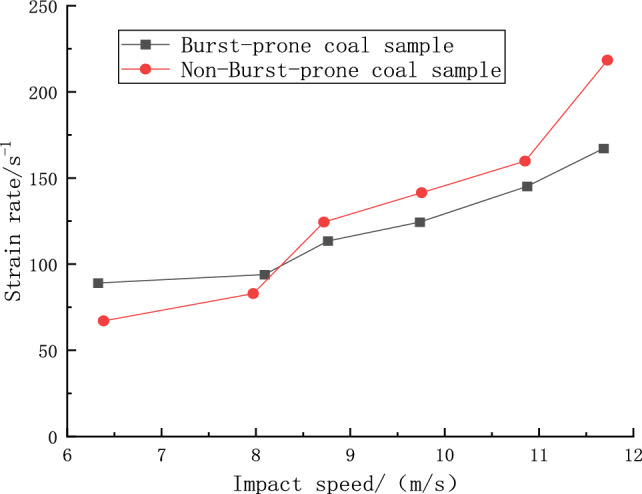
Relationship between impact velocity and strain rate.

Figure 8 delineates the correlation between the average strain rate and the bullet’s impact velocity for both groups of specimens. A discernible trend is that an augmented impact velocity engenders a notable increase in the strain rate for both sets of specimens. Specifically, within the impact velocity bracket of 6.31 m/s to 11.64 m/s, the impacted specimens experienced a surge in the average strain rate, growing from 87.76 to 168.83 s^−1^, or an enhancement of 192%. Conversely, the non-impacted specimens saw their average strain rate inflate even more drastically, from 67.43 to a substantial 220.28 s^−1^, marking a 327% increase. Analyzing the trends in the context of coal quality reveals that burst-prone coal specimens, characterized by their hard quality, manifested a smaller yet steadily increasing strain rate trajectory. In contrast, the non-burst-prone specimens, with their softer coal quality, exhibited higher sensitivity to strain and thus a more pronounced strain rate augmentation. This differentiation becomes particularly evident in the initial stages of the experiment. The non-burst-prone specimens, harboring a greater prevalence of minuscule cracks, necessitated a preliminary phase of compaction and deformation, with a portion of the imparted impact energy being channelized for crack compaction, resulting in a diminished initial strain rate. Notwithstanding, a critical point is reached at an impact velocity of 8.24 m/s. At this juncture, the growth trajectory of the non-burst-prone specimens overtakes that of their burst-prone counterparts, facilitated by a quicker crack compaction owing to heightened impact velocities. Consequently, at higher impact velocities, the non-burst-prone specimens sustain elevated strain rates compared to the burst-prone specimens, attributing to their intrinsic material properties and the dynamics of crack compaction accelerated by the increased impact velocity.

### Correlation between crushing work and strain rate

**Figure 9:**
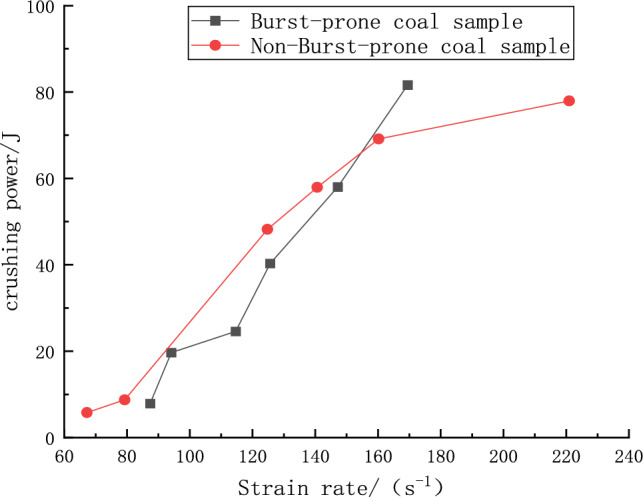
Variation of coal sample crushing work with strain rate.

In the course of dynamic compression trials conducted on two distinct sets of specimens, pertinent data on incident, reflected, and transmitted energies were garnered in line with the dynamic fluctuations observed throughout the experiments, leveraging the stress–strain waveforms discerned from SHPB experimentation procedure. This facilitated the computation of the crushing work pertinent to each set of specimens, achieved through the integration of the acquired data into Eq. (40). Subsequently, graphical representations delineating the progression of crushing work in relation to the strain rate over the span of the impact process were crafted, as visually encapsulated in Fig. 9.

Figure 9 elucidates a noticeable trend where the crushing work required to fracture the specimens escalates swiftly as the strain rate augments, evidencing a substantial strain rate growth effect. Despite the general similarities in the trajectory of the curves representing both specimen groups, distinct divergences in their respective increases are discernible. In specific terms, the impact specimen witnessed an augmentation in crushing work from 9.83 to 82.45 J over a strain rate interval of 87.76–168.83 s^−1^, culminating in an 8.39-fold surge. Conversely, the non-impact specimen experienced a more pronounced escalation, where the crushing work soared from 5.45 to 77.90 J across a strain rate span of 67.43–220.28 s^−1^, resulting in a 14.29-fold rise. This trend implicitly underscores a higher initial energy expenditure in the fracturing of the impact specimens as opposed to the non-impact ones. However, as the strain rate swells, the latter group exhibits a swifter proliferation in crushing work—a phenomenon aligning with the inherent fissure presence and more friable nature of the non-impact specimens, thereby necessitating a higher energy allocation for their disruption, and hence, a heightened crushing work.

## Discussion

### Fractal dimension theory and fractal dimension

In 1919, Hausdorff^41^ introduced the concept of dimensionality, demonstrating that it could be fractionalized through the application of fractal geometry; this fractionalized representation is referred to as the fractal dimension. The endeavor to harness fractal dimension theory centers around investigating the intricate structural attributes manifested in rock fragments of varying magnitudes and seemingly chaotic irregularities, with a focal point of uncovering the inherent fractal principles dictating the rock fracturing process.

During the fragmentation process, rocks delineate into fragments of disparate sizes. The customary approach to segregate these fragments involves utilizing sieves with divergent diameters *R*. Fragments exceeding the stipulated diameter progress to subsequent sieve levels, whereas those fitting the diameter criteria remain retained. This methodology gives rise to a characteristic relationship between the mass and frequency distribution of the fragmented rock components, as outlined below:

41 $$ \frac{m\left( R \right)}{m} = 1 - \exp \left[ { - \left( {\frac{R}{{R_{e} }}} \right)^{a} } \right] $$

where $m\left( R \right)$ is the sum of the masses of the crushed pieces of rock with sizes smaller than the diameter, in kg; $m$ is the total mass of the crushed pieces of rock, in kg; and *R*_*e*_ is the average particle size of the total crushed pieces.

Consequently, Eq. (16) can also be expressed as follows:

42 $$ \frac{{{\text{m}}\left( {\text{R}} \right)}}{{\text{m}}} = \left( {\frac{R}{{R_{e} }}} \right)^{a} $$

Taking the derivative of Eq. (17) as follows:

43 $$ dm \propto R^{a - 1} dR $$

Drawing upon the foundational principles of rock fractal theory, it can be deduced as follows:

44 $$ N\left( R \right) \propto R^{ - D} $$

45 $$ dN\left( R \right) \propto R^{D - 1} dR $$

where *N* is the total number of particles in the crushed mass of the rock.

There exists a specific relationship between the mass and the diameter, which is defined as follows:

46 $$ dm \propto R^{3} dN $$

Therefore the following expression can be obtained as follows:

47 $$ R^{a - 1} dR \propto R^{2 - D} dR $$

The fractional dimension number *D* of the fragmented pieces can be determined through rectification, as follows:

48 $$ D = 3 - a $$

where $a$ is the slope value of $\frac{m\left( R \right)}{m} - R$ in double logarithmic coordinates, which can be determined as follows:

49 $$ a = \lg \frac{m\left( R \right)}{m}/\lg R $$

### Fractal dimension of fractal dimension during coal fragmentation

**Figure 10:**
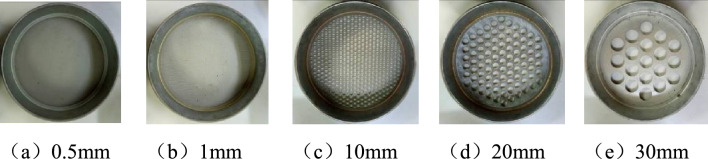
Coal sample sieve with different pore sizes.

Following the impact experimentation, all fragmented elements of the crushed specimens were meticulously gathered and stored in sealed bags, with distinct labels for identification. To categorize these fragments based on their sizes, the study utilized standard sieves with predefined apertures of 0.5 mm, 1 mm, 10 mm, 20 mm, and 30 mm, a selection informed by the initial dimensions of the specimens, which boasted a diameter and a length of 50 mm each. As illustrated in Fig. 10, this sieving process facilitated the segregation of fragment sizes, allowing for a detailed analysis of the distribution of various particle sizes present in the broken coal bodies from both groups. Subsequent to sieving, the fragments were weighed using a precision balance to attain a quantitative measure of their mass. By aggregating these data and computing the average mass for each sieve aperture category, the research crafted a comprehensive representation of the mass distribution among different sieve apertures, the details of which are expounded upon in Table 2.

**Table 2:** Mass distribution of different pore sieving fragments.

Specimen type	No	$\dot{\varepsilon }/s^{ - 1}$	Percentage of fractions screened with different apertures/%					
			< 0.5	0.5–1	1–10	10–20	20–30	> 30
Burst-prone coal sample	X-1	87.76	0.2	0.4	0.3	0.2	2.4	96.5
	X-2	94.32	1.1	0.2	5.8	2.3	1.4	89.2
	X-3	115.17	13.8	3.6	34.5	17.8	16.9	13.4
	X-4	125.26	16.6	4.8	38.4	20.1	12.6	7.5
	X-5	146.67	20.8	5.2	46.4	16.8	10.8	0
	X-6	168.83	27.0	7.2	53.1	8.6	4.1	0
Non-burst-prone coal sample	Y-1	67.43	0.1	1.2	41.8	48.9	4.3	3.7
	Y-2	80.37	0.3	4.7	48.2	41.6	3.1	2.1
	Y-3	124.45	0.4	3.9	55.8	36.4	2.2	1.1
	Y-4	139.39	3.6	3.9	60.9	31.5	0.1	0
	Y-5	160.27	4.5	5.2	67.7	22.6	0	0
	Y-6	220.28	4.1	6.4	72.8	16.7	0	0

**Figure 11:**
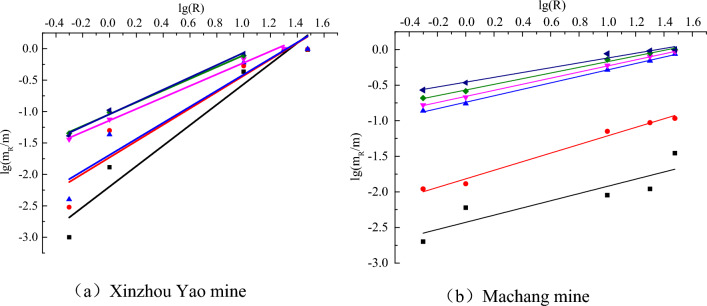
Fractal curves of two types of coal fragments.

The masses of fragments obtained from the various sieve apertures were accurately measured, facilitating the derivation of the proportion of mass present within each aperture interval through computational analysis. To further scrutinize the gathered data, a logarithmic transformation was applied, setting the stage for the construction of a double-logarithmic curve $\lg (m\left( R \right)/m) - \lg R$. Subsequently, a linear fit was applied to the transformed data to ascertain the slope *a* of the curve, which then paved the way for the determination of the fractal dimension D. Figure 11 visually portrays the derived fractal dimensions for both specimen groups, presenting a graphical comparison grounded in the comprehensive data sets. The slopes and correlation coefficients secured through the fitting of the fractal curve are meticulously cataloged in Table 3, offering a numerical depiction of the attributes observed in the fractal analyses corresponding to the diagrammatic representations in Fig. 11.

**Table 3:** Fractal dimension of coal samples.

Specimen type	No	Average fractal dimension D	Average correlation coefficient R^2^
Burst-prone coal sample	X-1	1.3688	0.9371
	X-2	1.6990	0.9634
	X-3	1.7207	0.9392
	X-4	2.0823	0.9367
	X-5	2.0232	0.9534
	X-6	2.1413	0.9424
Non-burst-prone coal sample	Y-1	2.3094	0.9794
	Y-2	2.3970	0.9564
	Y-3	2.4946	0.9672
	Y-4	2.5467	0.9438
	Y-5	2.5723	0.9578
	Y-6	2.6030	0.9962

### Experimental results and analysis

#### Fractional dimensional number and strain rate effect

**Figure 12:**
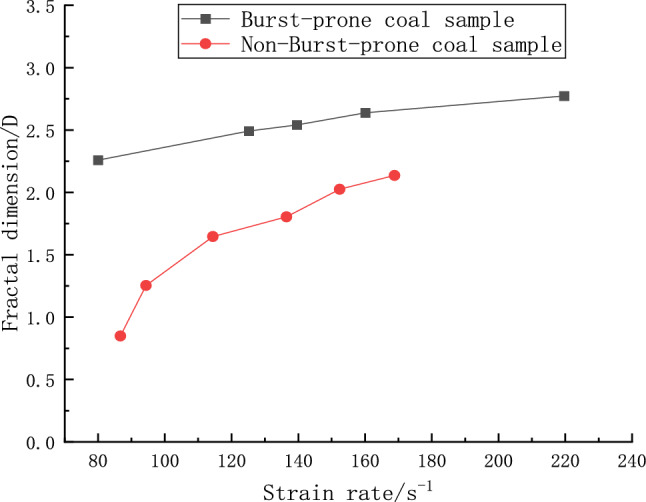
Relationship between fractal dimension and strain rate.

Figure 12 delineates the correlation between the fractal dimensions and the strain rates for both categories of coal specimens. It can be discerned from the data that there is a pronounced strain rate effect in play, with the fractal dimension escalating considerably as the strain rate amplifies. At a strain rate threshold of 80 s^−1^, the impacted coal specimen exhibits a fractal dimension value of 1.3688, markedly less than the 2.3094 characteristic of the non-impacted sample. This phenomenon reflects the intrinsically harder nature of the impacted sample, evidenced by its larger fragment sizes and consequently lower fractal dimensionality. Contrarily, the non-impacted sample, bearing more developed internal fissures and being looser, demonstrated a higher fractal dimensionality indicative of smaller fragment sizes. This aligns with prevailing scholarly insights into rock dynamics, affirming the theory that escalated impact loads engender finer rock fragments and heightened fractal dimensions. Furthermore, the graphical representation in Fig. 12 suggests a significant correspondence between strain rate and fractal dimension, albeit with nuanced dynamics at different strain rate stages. Below a strain rate of 100 s^−1^, non-burst-prone coal samples undergo more facile fragmentation, while the impacted specimens retain accumulated energy. Beyond this point, the fractal dimension for burst-prone samples surges exponentially, depicting an escalated fragmentation intensity. As strain rate increments further, a convergence in the fractal dimensions of both sample types is noted, approaching a critical value at elevated strain rates where the fragmentation peaks, resulting in largely homogenized fragment properties, resembling coal particles or even a powdery state. This trend potentially attributes to the inherent strength of burst-prone coal samples, which resist immediate drastic fragmentation, albeit absorbing increasingly more energy with augmented strength due to the sustained impact, ultimately yielding a greater fractal dimensionality in comparison to non-burst-prone specimens. This dynamic heralds elevated danger levels in scenarios of impact, spotlighting a critical aspect of material behavior under high strain rate conditions.

**Figure 13:**
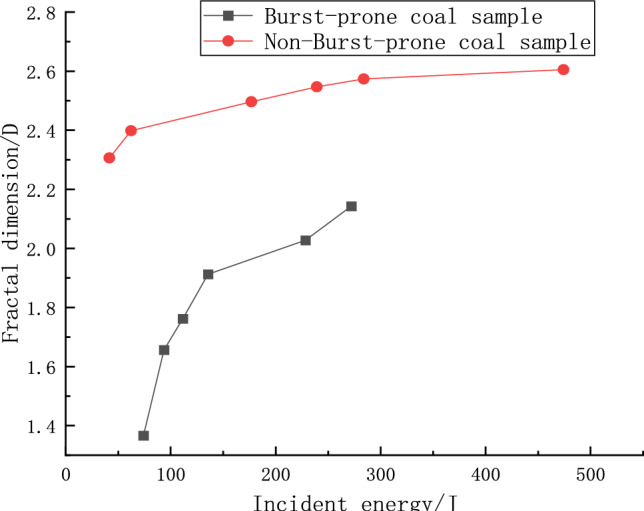
Relationship between fractal dimension and incident energy for two types of coal samples.

An analysis of the curves illustrating the fractal dimensionality and the incident energy for both categories of coal samples, as presented in Fig. 13, elucidates a notable rate correlation between these variables. As the incident energy escalates, there is a discernible increase in the fractal dimensional numbers for both sample types, albeit to varying extents. In the energy range of 0–50 J, the impacted coal sample exhibits a fractal dimension of 1.3688, while the non-impacted sample stands at 2.3094. This data suggests that within this specific energy interval, the non-impacted samples undergo more extensive fragmentation compared to their impacted counterparts, a phenomenon steered by the lower resilience and elastic–plastic properties intrinsic to the non-impacted samples. As the incident energy spectrum extends from 50 to 200 J, a rapid exponential augmentation is witnessed in the fractal dimensions of both sample types, marking increments by factors of 1.48 times and 1.11 times, respectively. The trend indicates diminishing fragment sizes for the impacted sample, a scenario likely spurred by the continual absorption of substantial energy levels, fostering a precipitous climb in the fractal dimension as the threshold of critical fragmentation nears. Upon attaining an incident energy benchmark of 300 J, a plateau is observed in the fractal dimensionality of the non-burst-prone samples, signifying complete fragmentation. Contrarily, the burst-prone samples exhibit a perpetuating increase in fractal dimensionality, attributed to two predominant factors: the innate hardness of the sample necessitating greater energy input for complete fragmentation, and the yet unreached peak of the impact energy spectrum, implying the necessity for further energy infusion to facilitate complete fragmentation.

#### Relationship between fractional dimension and crushing work

**Figure 14:**
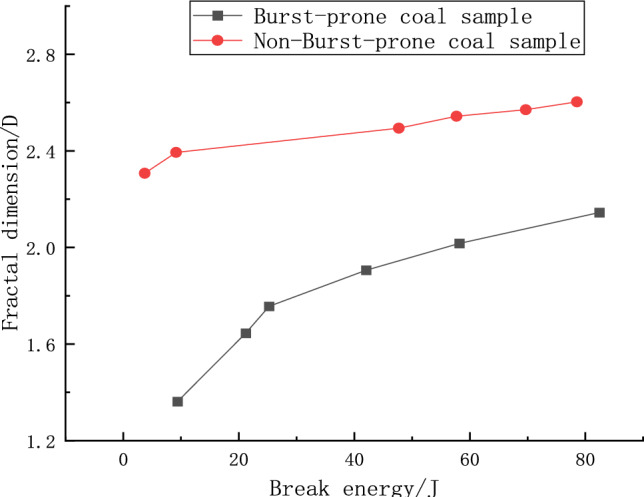
Relationship between fractal dimension and crushing work for two types of coal samples.

The correlation between the fractal dimension and the crushing work of the two coal specimen groups is delineated in Fig. 14. There exists a prominent rate correlation between the coal body crushing work and the fractal dimension, showcasing a trend where an increase in crushing work correlates to a respective augmentation in the fractal dimension to varying degrees. An analysis reveals that the absorbed energy—synonymous with crushing work—escalates as the impact strength augments, instigating a nearly linear rise in the fractal dimension of the coal specimens. In the initial stage where crushing work ranges from 0 to 20 J, the fractal dimensions for both impacted and non-impacted specimens mirror those observed at low strain rate conditions. This semblance serves to affirm the accuracy of the experimental crushing work data, attesting to minimal deviations. However, a distinct slowing in the rate of fractal dimension increase is observed as the crushing work approaches the 40 J mark. This phenomenon likely signifies the point at which the larger fragments of the coal body have predominantly been fractured, necessitating a greater amount of energy to break down the remaining smaller fragments, thereby entering a transient phase of heightened energy absorption. In comparison to the non-burst-prone coal specimens, the impacted specimens exhibit a greater extent of crushing work. This is attributable to their predisposition to absorb higher levels of energy as they are more susceptible to impact. Consequently, during this transitory phase of energy absorption, the impacted specimens manifest a swifter escalation in the fractal dimension.

### Characterization of damage morphologies

**Figure 15:**
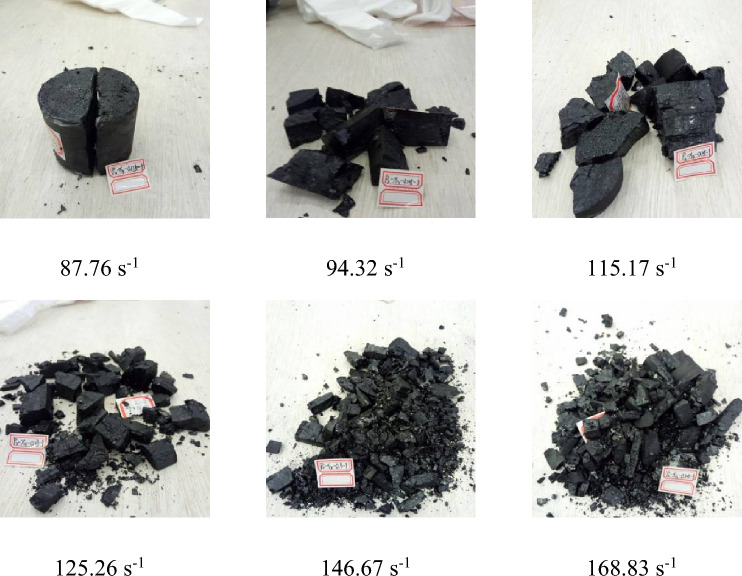
Failure modes of Xin zhou Yao Mine samples under different strain rates.

**Figure 16:**
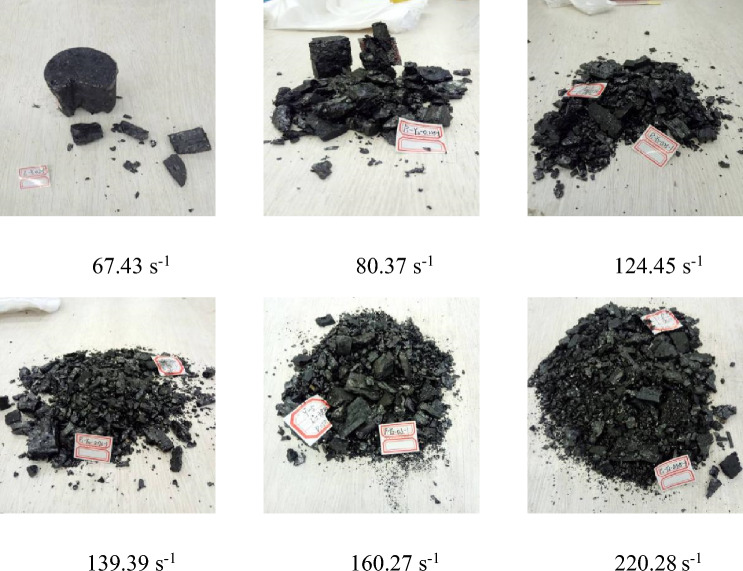
Failure modes of Ma Chang Mine samples under different strain rates.

Figures 15 and 16 illustrate the variations in damage morphologies sustained by two coal specimen groups under differing strain rates. A comprehensive analysis of these figures reveals that the coal specimens inevitably endure damage when subjected to impact loads, albeit with variations in the severity and nature of the damage conditioned by the strain rates in effect. The primary fracture directions serve as the predominant axes of extension and penetration for the ensuing damages. Notably, with a progressive increase in the force of the impact load, the strain rate governing the deformation of the coal specimens ceases to augment linearly. This irregularity in strain rate progression can be attributed to the expansion of internal fissures within the specimens, giving rise to amplified and uneven deformations. During the initial phases of this disruption, localized weak areas are the first to experience instability and succumb to damage. However, as strain rates continue to escalate, these fissures undergo further broadening and propagation, transforming localized damages into a more pervasive phenomenon. Consequently, the energy requisitioned for inducing specimen damage exhibits a proportional increase with the strain rate, intensifying the severity of the fragmentation into progressively finer particles. As the process approaches the material’s inherent limit for withstanding strain, an extreme scenario manifests—the specimens are pulverized into a powdery state, marking the zenith of destruction enabled by escalated strain rates.

An examination of Figs. 15 and 16 reveals a notable similarity in the damage patterns exhibited by the two sets of coal specimen groups, demonstrating a significant correlation with the strain rate. As the strain rate augments, a trend of intensified crushing and diminishing block size diameter is evident. Figure 16 delineates the damage morphologies sustained by impacted coal specimens across varying strain rates. In the strain rate interval of 87.76–115.17 s^−1^, the specimen manifests larger broken block sizes. This observation is underpinned by the intrinsic properties of the coal, directing the fracture development along the principal internal fissures. Given the hardness characteristic of the impacted coal specimens, a propensity for larger fragment sizes is witnessed at lower strain rates. Contrastingly, the same figure illustrates a substantially heightened degree of damage even at a strain rate of 80.37 s^−1^, progressing to near-total disintegration at a strain rate of 124.45 s^−1^. At this juncture, only minute fragments remain visible, a testament to the frail nature and diminished load-bearing capacity of the non-impacted specimens, rendering them more susceptible to breakage.

The mechanism underlying the damage to coal bodies fundamentally lies in the expansion of internal fissures driven by external forces. These fissures categorically fall into two groups: primary fissures, which are pre-existing, and regenerative fissures, emergent minute fissures birthed from the influence of impact loads. Upon the application of an impact load, a resultant stress wave propagates across the coal body specimen's surface, undergoing partial reflection while also partly infiltrating deeper layers of the specimen. This propagation engenders a tensile stress wave chiefly on the unrestrained surfaces adjacent to the main fracture extension. At lower strain rates, the damage pathway predominantly follows the axial extension, leading to fewer newly created fractures, thereby showcasing a tensile stress-focused damage. This phase witnesses a reliance on the expansion and deepening of existing primary fissures rather than the creation of new ones. However, with escalating impact loads, a pronounced development in newly formed fractures takes place. This phase sees the continuation of primary fissure extension, culminating in penetrating damage that is facilitated by the increasing synergistic activity between the proliferating fractures and the augmenting strain rate. As one ventures into higher strain rate regimes, the smaller fragments undergo heightened critical stress levels before succumbing to damage, necessitating the absorption of a greater quantum of energy.

## Conclusion

This study leveraged uniaxial compression experiments, utilizing the variable cross-section SHPB experimental setup, to scrutinize burst-prone and non-burst-prone coal samples under varying impact loads. The analysis facilitated an intricate understanding of the dynamic mechanical properties witnessed during the damage process, pivoting from an energy perspective and considering the sub-dimensionality of block degrees. Based on the insights derived from the energy dynamics and block degree sub-dimensions, the conclusions are reaching as follows:

1. The damage patterns exhibited by the two coal sample types diverge significantly when subjected to varying strain rate conditions. Under a uniform impact load, the impacted coal sample experiences fragmentation within the strain rate window of 87.76–115.17 s^−1^, resulting in larger debris fragments. These more substantial fragments necessitate a higher energy input for comprehensive disintegration, a requirement that escalates in direct proportion to increases in strain rate. In contrast, the non-impacted sample, characterized by a more friable structure, commences a higher degree of fragmentation at a strain rate of 124.45 s^−1^, with a predilection towards progressing into a powdered state as the strain rate amplifies.
2. The augmentation of impact velocity engenders a substantial growth effect in the incident, reflected, and transmitted energies across both sample categories, albeit with differing growth rates. Notably, the impacted sample displays a proclivity for more rapid energy concentration. The fragmentation process's crushing work exhibits a pronounced strain rate correlation, underscoring an escalating expansion and development of original fractures and the genesis of new ones. This trend delineates a distinctive growth pace for burst-prone samples, demonstrating a quicker response trajectory compared to their counterparts.
3. Increasing strain rates witness a consistent escalation in the fractional dimensional numbers of fragments for both coal samples under the purview of impact loads. This signifies that heightened fragmentation degrees parallel a reduction in fragment particle sizes, progressing towards a powdered state. Furthermore, a conspicuous growth effect materializes in correlation with incident energy and crushing work, as evinced by the behavior of the fractional dimensional number.

## Data Availability

The raw data supporting the conclusion of this article will be made available by the authors, without undue reservation. Xiao-He Wang (wangxh_1994@163.com) should be contacted if someone wants to request the data from this study.
